# The Arf-like GTPase Arl8b is essential for three-dimensional invasive growth of prostate cancer *in vitro* and xenograft formation and growth *in vivo*

**DOI:** 10.18632/oncotarget.8832

**Published:** 2016-04-18

**Authors:** Samantha S. Dykes, Alana L. Gray, David T. Coleman, Madhurima Saxena, Charles A. Stephens, Jennifer L. Carroll, Kevin Pruitt, James A. Cardelli

**Affiliations:** ^1^ Department of Microbiology and Immunology, LSU Health Shreveport, Shreveport, LA, USA; ^2^ Feist-Weiller Cancer Center, LSU Health Shreveport, Shreveport, LA, USA; ^3^ Department of Molecular and Cellular Physiology, LSU Health Shreveport, Shreveport, LA, USA; ^4^ Current address: Department of Medical Oncology, Dana-Farber Cancer Institute and Department of Medicine, Harvard Medical School, Boston, MA, USA; ^5^ Current address: Texas Tech University Health Sciences Center, Lubbock, TX, USA

**Keywords:** Arl8b, lysosome, invasion, xenograft, lipid metabolism

## Abstract

Cancer is a multistep process that requires cells to respond appropriately to the tumor microenvironment, both in early proliferative stages and in later invasive disease. Arl8b is a lysosome localized Arf-like GTPase that controls the spatial distribution of lysosomes via recruitment of kinesin motors. Common features of the tumor microenvironment such as acidic extracellular pH and various growthfactors stimulate lysosome trafficking to the cell periphery (anterograde), which is critical for tumor invasion by facilitating the release of lysosomal proteases to promote matrix remodeling. Herein we report for the first time that Arl8b regulates anterograde lysosome trafficking in response to hepatocyte growth factor, epidermal growth factor, and acidic extracellular pH. Depletion of Arl8b results in juxtanuclear lysosome aggregation, and this effect corresponds with both diminished invasive growth and proteolytic extracellular matrix degradation in a three-dimensional model of prostate cancer. Strikingly, we found that depletion of Arl8b abolishes the ability of prostate cancer cells to establish subcutaneous xenografts in mice. We present evidence that Arl8b facilitates lipid hydrolysis to maintain efficient metabolism for a proliferative capacity in low nutrient environments, suggesting a likely explanation for the complete inability of Arl8b-depleted tumor cells to grow in vivo. In conclusion, we have identified two mechanisms by which Arl8b regulates cancer progression: 1) through lysosome positioning and protease release leading to an invasive phenotype and 2) through control of lipid metabolism to support cellular proliferation. These novel roles highlight that Arl8b is a potential target for the development of novel anti-cancer therapeutics.

## INTRODUCTION

The formation of metastatic foci from an invasive primary tumor is responsible for nearly all instances of cancer-associated death. In general, progression to metastasis requires cancer cells be amenable to adjusting their metabolic phenotype in response to the ever-changing tumor microenvironment (TME). During later stages in the metastatic cascade, tumor cells must acquire the ability to remodel the extracellular matrix (ECM) through secretion of proteases, allowing for invasion and metastasis. Numerous published studies suggest lysosomes can profoundly influence these processes (reviewed in [1]).

The role of lysosomes in tumor progression is complex and consists of three notable functions: 1) Following fusion with lipid inclusion bodies, lysosomes play a role in the hydrolysis of stored and internalized neutral lipids through lysosomal acid lipase (LAL) activity [2, 3]; 2) Lysosome trafficking has been linked to nutrient-sensing signaling cascades involving mammalian target of rapamycin (mTOR) activity and autophagy [4]; 3) Lysosomes supply proteases for ECM degradation that can lead to invasion and metastasis [1]. Each of these lysosome functions is dependent on intracellular lysosome mobility along cytoskeletal components.

Lysosomes are acidic organelles that normally function as the terminal degradative end point of the endocytic pathway. As such, lysosomes are typically found in the perinuclear region of quiescent cells. Our laboratory and others have determined that stimuli commonly found in the TME influence lysosome positioning within cells. Acidic extracellular pH (pH_e_) and growth factor signaling drive anterograde (plus end or peripheral) lysosome trafficking, and we have previously demonstrated that the spatial distribution of lysosomes within tumor cells dictates secretion of cathepsin B and invasiveness [5–8]. Cathepsin B is an important protease that regulates tumor cell invasion, and lysosomes proximal to the plasma membrane facilitate the release of cathepsins to the extracellular environment [9]. Although evidence suggests that some forms of tumor cell invasion are protease independent, a growing body of data indicates that lysosomal cathepsins and matrix metalloproteinases (MMPs) are major contributors to invasion *in vitro* and *in vivo* [1, 10–12]. Blocking lysosome trafficking to the plasma membrane results in decreased protease secretion and reduced invasion [5–8].

Lysosome trafficking along microtubules is mediated by dynein and kinesin motors in a retrograde (minus end or toward the microtubule organizing center (MTOC)) or anterograde fashion, respectively [13–15]. Several GTPases are known to regulate the recruitment of kinesins and dyneins to lysosomes. For example, the lysosome-localized GTPase Rab7 is well-known for its role in recruiting dyneins to lysosomes through its effector Rab-interacting lysosomal protein (RILP) [14], and we have recently established a role for Rab7 as a potential tumor suppressor via its ability to cluster lysosomes near the MTOC [8].

ADP-Ribosylation Factor like Protein 8b (Arl8b) is an Arf-like GTPase that when in the GTP-bound, activated state is specifically localized to lysosomes and controls lysosome positioning within the cell via recruitment of motor proteins [16, 17]. Arl8b recruits kinesin 1 to lysosomes to promote anterograde lysosome trafficking [18]. Loss of Arl8b or expression of an Arl8b inactive mutant, results in tight clustering of lysosomes over the MTOC [16]. Arl8b has also been implicated in lysosome fusion, immune cell function, and lysosomal tubulation [19–23]. Arl8b can be recruited to lysosomes in response to ErbB2 signaling, supporting a mechanism for lysosome redistribution in response to cancer-associated extracellular stimuli [24]. Recent reports suggest that Arl8b regulates cell motility and cell spreading [23, 25]; however, Arl8b has not yet been investigated in the context of tumor growth and invasion.

Herein, we analyzed whether Arl8b plays a role in tumor progression. We found that Arl8b is required for invasion and protease secretion in 3D culture and report that Arl8b is required for prostate tumor growth in a xenograft mouse model. Interestingly, Arl8b depletion does not affect proliferation in complete growth media, but greatly impairs proliferation in the absence of serum which appears to be associated with an aberrant lipogenic phenotype. Overall, these data suggest Arl8b is a potential target to prevent prostate cancer progression.

## RESULTS

### Depletion of Arl8b prevents anterograde lysosome trafficking in response to acidic pH_e_ and growth factors

**Figure 1:**
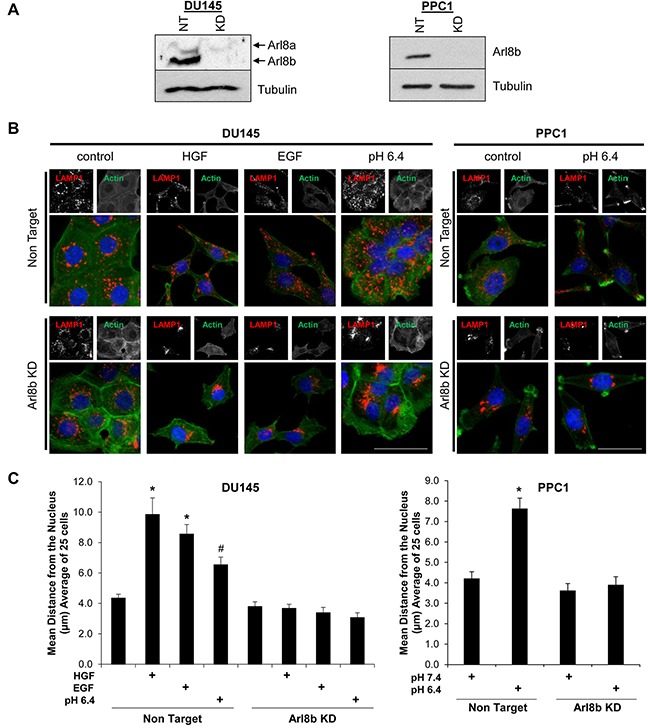
Arl8b knockdown prevents low pH-, EGF-, and HGF-induced lysosome trafficking to the cell membrane **A.** DU145 and PPC1 cells were transduced with lentiviral-delivered shRNA sequences targeted to Arl8b (KD) or non-targeted (NT) shRNA. Immunoblots confirm knockdown. **B.** DU145 and PPC1 cells were treated with low pH for 2 hours, or 33 ng/mL HGF or 100 ng/mL EGF for 18 hours then fixed, stained for LAMP-1 (Red), phalloidin (Green), and DAPI (Blue). **C.** Quantitated lysosome distribution from 25 cells shown as mean ± SEM; *=p<0.001 compared to NT control, #=p<0.05 compared to NT control.

Previous studies have identified Arl8b as a key regulator of lysosome spatial distribution [16, 17]. We have previously demonstrated that lysosomes traffic toward the plasma membrane in response to acidic pH_e_, hepatocyte growth factor (HGF), or epidermal growth factor (EGF) found within the TME [6, 7]. To determine whether Arl8b plays a role in this stimulus-driven anterograde lysosome redistribution, we transduced DU145 and PPC1 human PCa cell lines with Non Target (NT) or Arl8b-targeted lentiviral-delivered shRNA to generate stable cell lines. Immunoblot analysis revealed that Arl8b protein levels were depleted by more than 90% in DU145 and PPC1 cells (Figure 1A). NT and Arl8b KD cells were treated with serum-free media containing HGF or EGF, or serum-free media at pH 6.4 for 18 hours and stained for LAMP-1 (a marker for late endosomes and lysosomes) (Figure 1B). The lysosomes in DU145 cells responded to all conditions, while lysosomes in PPC1 cells did not traffic in response to growth factors (data not shown). In accordance with previously published literature, lysosomes in DU145 and PPC1 NT cells underwent anterograde lysosome trafficking upon treatment with acidic media and lysosomes were found near the plasma membrane. In contrast, DU145 and PPC1 Arl8b KD cells maintained lysosomes significantly closer to the nucleus compared to NT cells (Figure 1B; quantified in Figure 1C). The knockdown and lysosome phenotype were confirmed with additional shRNA clones for both DU145 (Figure S1A, S1B) and PPC1 cells (Supplementary Figure S1C, S1D). We also generated Arl8b KD in MDA MB 231 human breast cancer cells (Supplementary Figure S1E) and confirmed Arl8b KD prevents acidic pH_e_-mediated anterograde lysosome trafficking in breast cancer cells as well (Supplementary Figure S1F). Overall, these data suggest that Arl8b is necessary for the redistribution of lysosomes in response to defined external stimuli that are commonly found in the TME.

### Kif5b is necessary for lysosome trafficking in response to acidic pH_e_ and growth factors

Arl8b is known to promote anterograde lysosome trafficking via recruitment of kinesin 1 to lysosome membranes [18]. Kinesin 1 drives plus-end trafficking, and previous reports indicate that depletion of the kinesin 1 heavy chain (Kif5b) results in a tight clustering of lysosomes near the nucleus in embryonic mouse cells [15]. We next tested whether Kif5b was necessary for lysosome dispersion in response to acidic pH_e_ and growth factors. To this end, we depleted Kif5b using lentiviral-delivered shRNA and confirmed knockdown by immunoblot (Supplementary Figure S2A). NT and Kif5b KD cells were stimulated with acidic pH_e_, HGF, or EGF and lysosome positioning was observed by immunofluorescence (Supplementary Figure S2B). In line with our Arl8b KD findings, Kif5b depletion prevented anterograde lysosome trafficking in response to acidic pH_e_ and growth factor stimulation, validating the cooperative function of Arl8b with kinesin 1.

### Arl8b KD prevents protease secretion and invasion in a 3D culture model

**Figure 2:**
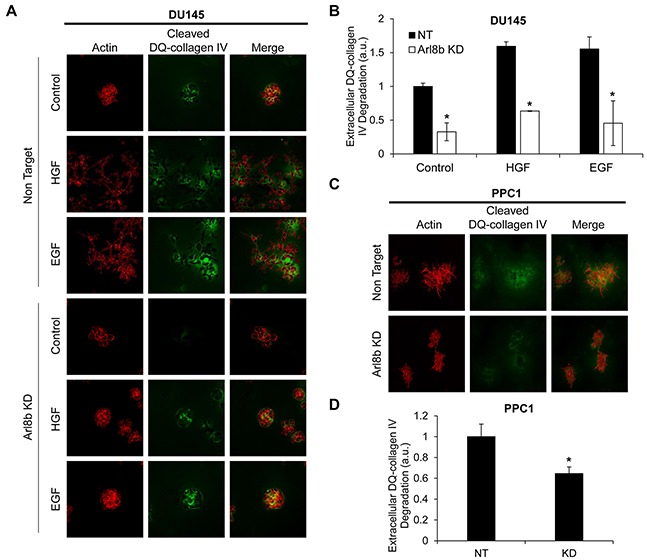
Arl8b knockdown prevents the formation of invasive outgrowths and matrix degradation **A.** DU145 and **C.** PPC1 cells were grown in Matrigel and DQ-Collagen IV for 72 hours under the indicated experimental conditions then fixed and stained with phalloidin (red). Green represents DQ-collagen IV cleavage as a readout for protease secretion. Images are representative of three independent experiments and are quantified in **B.** and **D.**. Data are shown as mean ± SEM;*=p<0.05 compared to NT.

We have previously described the role of lysosome distribution in tumor cell invasion and cathepsin B secretion and found that the lysosomal GTPase Rab7, by promoting perinuclear lysosome localization, is a potential tumor suppressor [8]. However, the role of Arl8b in tumor cell invasion and protease secretion has not been previously investigated. We used a Matrigel 3D culture model with embedded DQ-collagen IV to assess the necessity of Arl8b for cellular invasion and protease activity. DQ-collagen fluoresces upon proteolytic cleavage and indicates ECM degradation by proteases, including those released from lysosomes [26, 27]. DU145 or PPC1 NT and Arl8b KD cells were grown in 3D culture for two days followed by an additional two days of stimulation with HGF or EGF in serum-free media (Figure 2A; quantified in Figure 2B). Protease activity, as indicated by cleaved DQ-collagen IV fluorescence, was increased in DU145 NT cells treated with growth factor compared to NT control cells. This increased protease activity was accompanied by the formation of invasive structures and loss of spheroid colony morphology. Both the formation of invasive outgrowths and protease secretion were prevented by Arl8b depletion. Unlike DU145 cells, PPC1 cells form large branching colonies with copious protease activity even in the absence of growth factor. However, when Arl8b is knocked down, PPC1 cells form smaller, more compact colonies with minimal protease secretion and few invasive outgrowths (Figure 2C; quantified in Figure 2D). These studies indicate that Arl8b is necessary for prostate tumor cell protease release, matrix degradation, and tumor cell invasion in a 3D environment.

### Loss of Arl8b does not impair growth factor signaling or cell scattering

**Figure 3:**
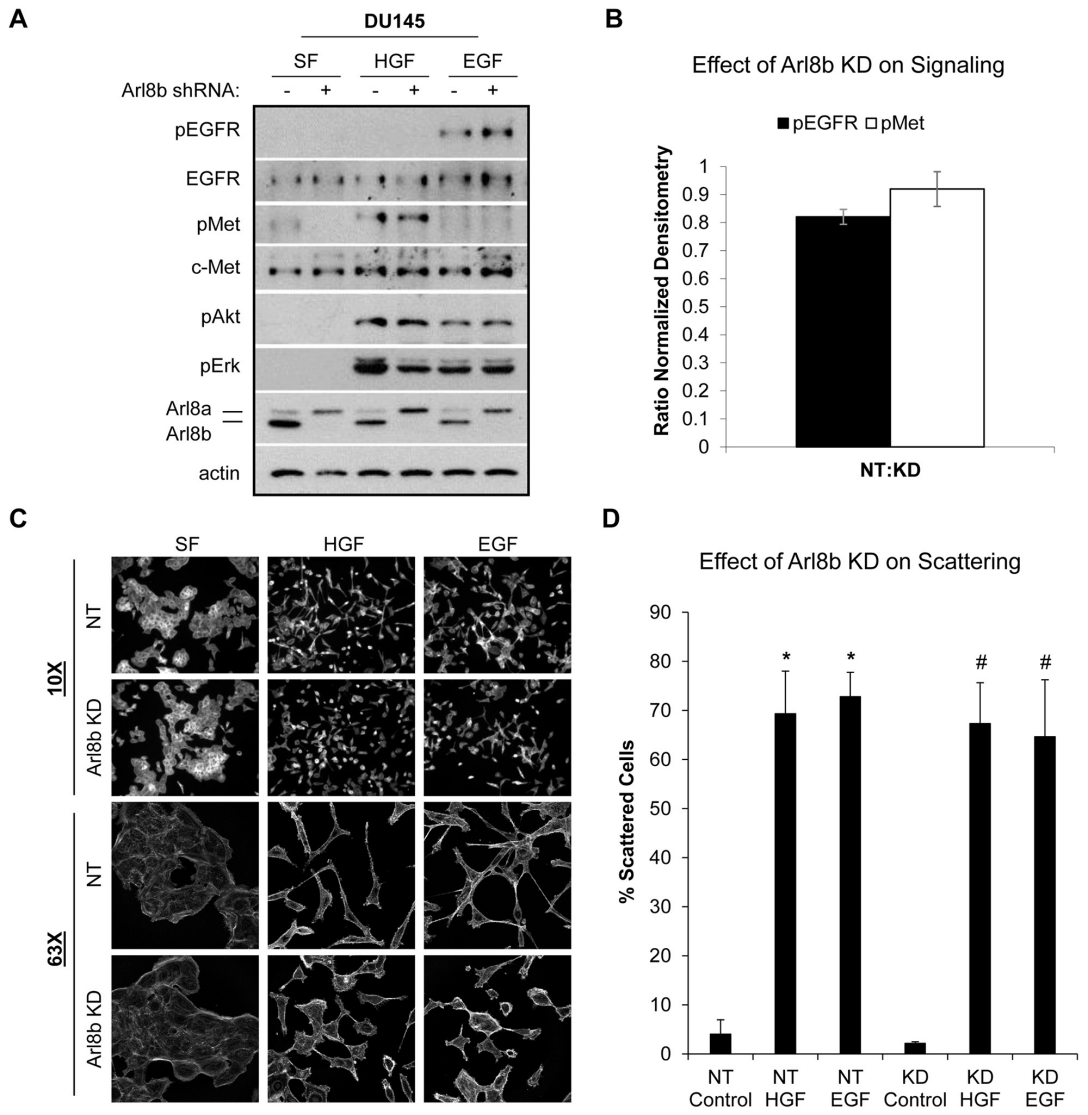
Arl8b knockdown does not affect HGF- or EGF-induced signaling or cell scattering **A.** DU145 NT and Arl8b KD cells were serum starved 30 minutes prior to treating with 33 ng/mL HGF or 100 ng/mL EGF for 20 minutes. Cell lysates were analyzed by immunoblot for the indicated proteins. **B.** Ratio of normalized densitometry from three independent immunoblots. Data are shown as mean ± SEM. **C.** DU145 NT and Arl8b KD cells were treated with 33 ng/mL HGF or 100 ng/mL EGF overnight in serum-free media. Cells were fixed and stained for actin. Representative images from 10X and 63X fields are shown, N=3. **D.** Quantitative analysis of cell scattering from three independent experiments. Data are shown as mean ± SEM; *=p<0.001 compared to NT control and #=p<0.001 compared to KD control.

HGF and EGF signaling are often hyperactivated in cancer, culminating in increased motility and invasion (reviewed in [28, 29]). Previous studies have identified the lysosomal GTPase Rab7 as a regulator of both EGFR and c-Met signaling via the control of lysosome-mediated receptor degradation [8, 30]. Immunoblot analysis of DU145 NT and Arl8b KD cells treated with HGF or EGF revealed that the absence of Arl8b does not prevent activation of the c-Met or EGF receptors or downstream signaling (Figure 3A; quantified in Figure 3B). DU145 PCa cells respond to long term EGF and HGF stimulation by losing cell-cell adhesions and undergoing an epithelial to mesenchymal transition (EMT) whereby cells take on a scattered appearance [31]. To test whether Arl8b depletion disrupted the motile response to growth factor signaling, DU145 NT and Arl8b KD cells were treated with HGF or EGF and scattering was assessed by fluorescence microscopy (Figure 3C; quantified in Figure 3D). Although Arl8b KD cells still scattered in the presence of growth factors, these cells appeared shorter than their elongated NT counterparts, suggesting that loss of Arl8b and/or juxtanuclear lysosome aggregation may alter cell spreading and actin dynamics. Additionally, time-lapse microscopy revealed that both DU145 and PPC1 Arl8b KD cells had delayed cell spreading and attachment compared to NT cells (Supplementary Figure S3). Although Arl8b depletion does not prevent an EMT phenotype, there was an apparent disparity in morphology that suggests altered actin dynamics.

### Active Rac1 and RhoA levels are decreased in Arl8b KD cells

Published reports indicate that the position of lysosomes can contribute to the spatial regulation of proteins controlling actin dynamics including GTPases RhoA and Rac1, thus influencing leading edge morphology and cell motility [32–35]. Due to the stunted cellular phenotype (Figure 3C) and the 3D invasion results (Figure 2), we investigated RhoA and Rac1 activity in response to Arl8b knockdown. DU145 NT and Arl8b KD cells were treated with or without EGF, and Rac1 or RhoA activity were assessed by GST-PAK1 or RhoA-Rhotekin pulldown, respectively. Immunoblot analysis revealed that basal active Rac1 and RhoA levels were significantly decreased in Arl8b KD cells compared to NT cells (Supplementary Figure S4A, S4C), although EGF-stimulated Rac1 and RhoA activity was not significantly different in Arl8b KD cells (Supplementary Figure S4B, S4D). These data indicate that loss of Arl8b results in modestly reduced basal levels of active Rac1 and RhoA, but does not hinder their activation potential by growth factor. Therefore, the decrease in Rac1 and RhoA activity is unlikely to account for the reduced invasion (Figure 2) and defective cell spreading observed in Arl8b KD cells when stimulated with growth factor (Figure 3C and Supplementary Figure S3).

### Co-culture with NT cells does not rescue motility defects seen in Arl8b KD cells

**Figure 4:**
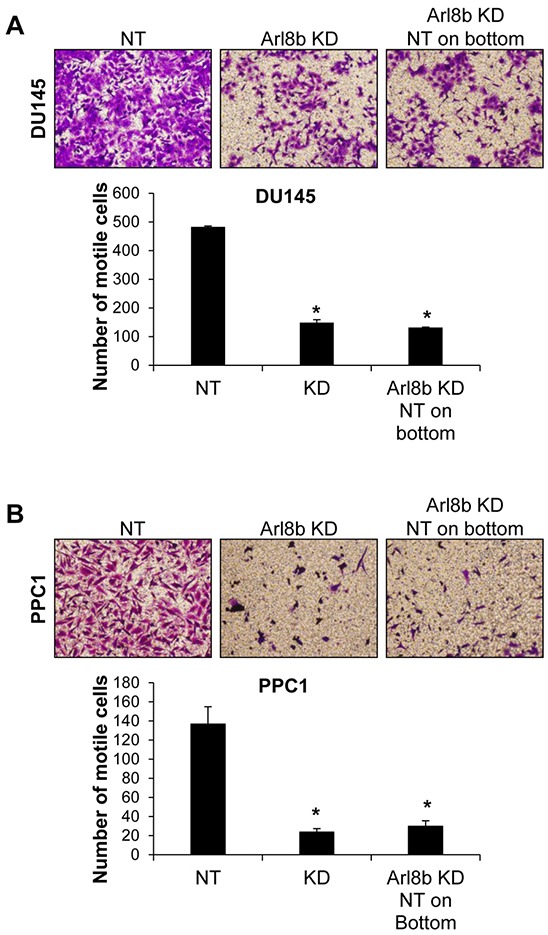
Defective Arl8b KD motility is not rescued by co-culture with NT cells **A.** DU145 or **B.** PPC1 cells were grown on top of transwell inserts in serum-free media and allowed to migrate toward serum-containing media for 48 hours. Cells were fixed and stained with crystal violet. Images represent 10X fields of cells that have migrated to the underside of the insert, N=3. Graphs represent the average number of motile cells from three independent experiments; *=p<0.001 compared to NT.

Given that Arl8b is involved in vesicle trafficking, it is possible that knockdown prevents the secretion of a protein(s) necessary for motility. Accordingly, we tested whether co-culture with NT cells could rescue the phenotype by supplying this hypothetical protein in *trans*. We performed transwell motility assays with both DU145 (Figure 4A) and PPC1 cells (Figure 4B). NT or Arl8b KD cells were seeded on the top of the transwell insert and allowed to migrate toward complete media on the underside of the insert. Arl8b KD cell migration toward complete media was less efficient compared to NT. Alternatively, NT cells were plated on the well underneath the insert and Arl8b KD cells were seeded on top. Contact with conditioned media from the NT cells did not rescue Arl8b KD motility to levels similar to NT. Together, these data indicate Arl8b depletion prevents cell motility by restricting lysosome trafficking in a manner not able to be supplanted in *trans*.

### Arl8b knockdown prevents tumor growth *in vivo*

**Figure 5:**
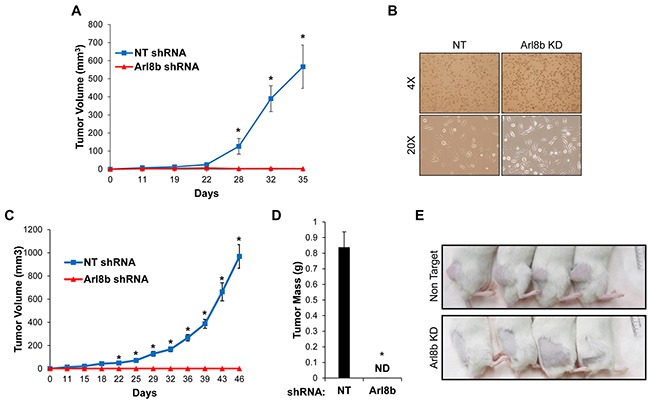
Arl8b depletion prevents tumor growth *in vivo* **A.** PPC1 NT or Arl8b KD cells were grown subcutaneously in SCID/bg mice and tumor volume was measured over time. Data are shown as mean ± SEM; *=p<0.001 compared to Arl8b shRNA. **B.** Immediately following subcutaneous injections, remaining cell suspensions were directly seeded onto 10 cm dishes through the same needle and syringe used for injections and allowed to settle for 24 hours in complete media. **C.** PPC1 NT or Arl8b KD cells were grown subcutaneously in SCID/bg mice and tumor volume was measured over time. Data are shown as mean ± SEM; *=p<0.001 compared to Arl8b shRNA. **D.** Tumor weight was measured at time of harvest. Data are shown as mean ± SEM; *=p<0.001 compared to NT shRNA. **E.** Representative mice from each experimental group are shown.

Due to the strong effect of Arl8b silencing on tumor cell invasion in 3D Matrigel (Figure 2), we sought to determine the necessity of Arl8b for tumor progression *in vivo*. PPC1 PCa cells were used in a xenograft model because of their propensity to form tumors. 1×10^6^ NT or Arl8b KD PPC1 cells were injected subcutaneously into the hind flank of SCID/bg male mice and tumor growth was measured twice-weekly using a caliper (Figure 5A). The results from this initial experiment showed a striking difference in tumor growth between NT and Arl8b KD cells. In repeating the experiment, at the time of subcutaneous injection, cell culture plates were seeded in parallel from the cell suspensions prepared for injection to assay for possible differences in sensitivity to the preparation. Remaining cells from the injection prep were passed through a needle to mimic injection and cultured under normal *in vitro* conditions (Figure 5B). Microscopic examination revealed that both NT and Arl8b KD cells attached to the plastic dish, had similar numbers of cells per field, and were viable 24 hours later, indicating that cells did not die during the preparations for *in vivo* experiments. Interestingly, the NT tumors grew at an exponential rate, while Arl8b KD cells did not form tumors and at no point were detectable by palpation (Figure 5C and 5D). Representative images of mice clearly show the substantial growth of NT cell xenografts and the complete absence of tumor formation from Arl8b shRNA expressing cells (Figure 5E). In fact, necropsy examination of the injection site revealed no sign of cancer cell growth. The lack of tumor growth from Arl8b depleted cells indicates an essential role for Arl8b in tumor seeding and growth initiation within an *in vivo* environment.

### Arl8b knockdown impairs proliferation under limited nutrient conditions

Cancer stem cells are thought to drive tumor initiation [36]. Given the inability of Arl8b depleted cells to initiate tumor growth *in vivo*, we sought to determine whether Arl8b knockdown resulted in a depleted stem cell population. To assess this possibility, we collected whole cell lysates from DU145 and PPC1 cells expressing NT or Arl8b shRNA and probed for prostate cancer stem cell markers, CD44 and ALDH1A1, by immunoblot (Supplementary Figure S5A) [37, 38]. We found that CD44 and ALDH1A1 protein expression was not reduced in Arl8b KD cells for either cell line, suggesting that the inability of Arl8b KD cells to establish tumors *in vivo* is likely not the result of a diminished stem cell population.

**Figure 6:**
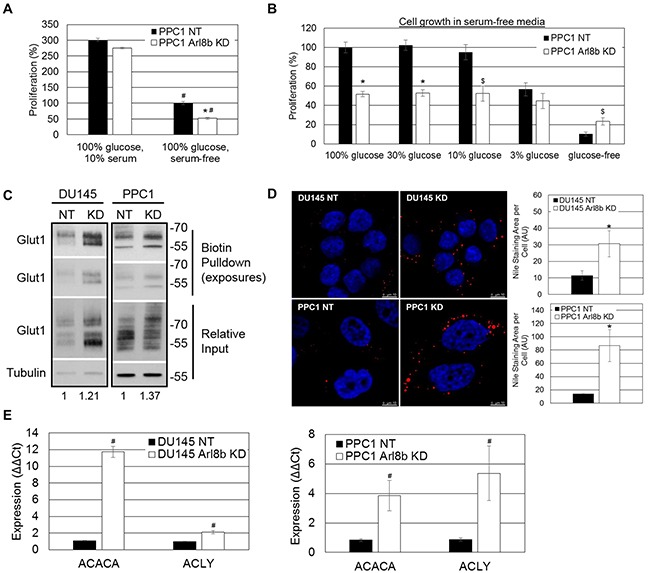
Arl8b depletion imposes an aberrant lipogenic phenotype to prostate cancer cells and impairs proliferation under limited nutrient conditions **A.** PPC1 NT and Arl8b KD cells were seeded to approximately 15% confluency prior to proliferation analysis in complete growth media (10% serum, 100% glucose). Data are shown as mean ± SEM; *=p<0.05 compared to NT serum-free and #=p<0.01 compared to cells grown in complete media, or **B.** serum-free media at 100%, 30%, 10%, or 3% of normal glucose concentration. Relative change in confluency over 4 days was determined using the IncuCyte ZOOM imaging system and analysis software. Conditions were tested in quadruplicate for three independent experiments. Data are shown as mean ± SEM; *=p<0.01 compared to NT and $=p<0.05 compared to NT. **C.** Biotinylated cell surface protein from DU145 or PPC1 NT and Arl8b KD cells growing in complete growth media was isolated and immunoblotted for Glut1. Densitometric analysis of Glut1 expression relative to Tubulin is shown. **D.** DU145 or PPC1 NT and Arl8b KD cells serum starved for twelve hours were fixed and stained for neutral lipid inclusion bodies. Representative images from 63x-objective confocal microscopy are shown and quantitation from 40x-objective confocal fields (five fields, three independent experiments) is graphed. Data are shown as mean ± SEM; *=p<0.01 compared to NT. **E.** RNA from DU145 or PPC1 NT and Arl8b KD cells grown in complete media was analyzed by qPCR for acetyl CoA carboxylase (ACACA) and ATP citrate lysase (ACLY). Data are shown as mean ± SEM; *=p<0.01.

The subcutaneous injection site, like an established TME, is characterized by poor blood flow and reduced nutrient availability [39–41]. To determine whether Arl8b depletion hinders efficient nutrient usage and lessens growth in a nutrient-poor environment, we assessed proliferation in the absence of serum and under reduced glucose concentrations. When grown in complete media, PPC1 NT and Arl8b KD cells had similar rates of proliferation (Figure 6A). However, in the absence of serum there was a 50% difference in growth rate between NT and Arl8b KD cells (Figure 6A, 6B). In fact, by serially restricting glucose levels, we found that NT cell growth was not impaired to the level of Arl8b KD cells until glucose levels were reduced to 3% of normal levels (100%) (Figure 6B). These data indicate that Arl8b knockdown causes a defect in metabolism required for cell proliferation. Given that Arl8b KD cells are unable to proliferate in low nutrient conditions, these data may partially explain the inability to establish tumors *in vivo*.

### Arl8b depletion imposes an aberrant lipogenic phenotype to prostate cancer cells

Previous reports suggest that Arl8b and lysosome positioning are involved in controlling cellular nutrient response and mammalian target of rapamycin complex 1 (mTORC1) activation [4]. The inability to respond to low nutrient conditions could contribute to the lack of growth of Arl8b KD cells *in vivo* and *in vitro* in the absence of serum. To test this, PPC1 cells were treated with media containing varying concentrations of serum. Rapamycin, a known inhibitor of mTOR, was used as a control. Whole cell lysates were collected and probed for phospho-p70 s6 kinase (activated by mTOR) by immunoblot analysis (Supplementary Figure S5B). PPC1 Arl8b KD cells had similar levels of p70 s6 kinase phosphorylation compared to their respective NT controls, supporting an equivalent ability to sense nutrient deprivation through the mTOR pathway.

To account for impaired proliferation in low glucose conditions, it is possible that Arl8b depletion prevents sufficient trafficking of the glucose transporter Glut1 to the plasma membrane [42]. To test this, we performed surface biotinylation to assess the expression of Glut1 on DU145 or PPC1 NT and Arl8b KD cells. Immunoblot analysis from biotin pulldown samples indicated that there were slightly elevated levels of Glut1 surface expression when Arl8b was depleted (Figure 6C). These results suggest Arl8b KD cells are equally capable of glucose uptake compared to NT cells.

Given the impaired proliferative phenotype, despite adequate Glut1 surface expression, we hypothesized that impaired proliferation under nutrient-poor conditions *in vivo* and *in vitro* is due to an inability to hydrolyze internalized lipids by lysosomal hydrolases [43]. To test this we stained DU145 and PPC1 NT and Arl8b KD cells for neutral lipids after serum removal for 12 hours. Quantitation of neutral lipid staining indicated a large increase in the abundance of intracellular neutral lipid inclusion bodies upon Arl8b KD suggesting impaired neutral lipid hydrolysis (Figure 6D). These results mirror Wolman's disease and Niemann Pick type C and D in which impaired liberation of neutral lipids by lysosomes, due to hydrolase deficiency or blocked lipid transport, leads to accumulation in inclusion bodies [2, 44, 45].

Dysfunctional lipid hydrolysis can cause an aberrant decrease in intracellular lipids and heightened sterol regulatory element-binding protein (SREBP) transcriptional activity resulting in constitutively elevated lipogenic gene expression [2, 44, 45]. To test whether Arl8b depletion, causing impaired lysosomal mobility and/or lysosome-endosome fusion, leads to a constitutively elevated lipogenic phenotype, we analyzed expression of SREBP-responsive genes acetyl CoA carboxylase (ACACA) and ATP citrate lyase (ACLY). For both DU145 and PPC1 cells, when Arl8b is depleted, ACACA and ACLY expression are elevated, indicating increased SREBP transcriptional activity (Figure 6E). These data taken together support a critical role for Arl8b in cellular metabolism that is necessary for efficient glucose utilization and a proliferative phenotype.

## DISCUSSION

The results presented herein indicate two critical roles for Arl8b in cancer progression through control of lysosome mobility or fusion. 1) We have found for the first time that in the absence of Arl8b, cancer cells exhibit restricted anterograde lysosome trafficking and, thus, reduced release of lysosomal proteases thereby preventing ECM degradation and invasive growth. 2) We have found that depletion of Arl8b impairs hydrolysis of internalized and/or stored neutral lipids thereby shifting the metabolic profile toward an aberrant lipogenic phenotype resulting in inefficient glucose utilization, limiting the propensity for cell division. In fact, upon loss of Arl8b, prostate cancer cells were absolutely unable to grow as xenograft tumors.

Our laboratory and others have demonstrated that trafficking of lysosomes toward the plasma membrane is promoted by a number of stimuli present in the TME, including growth factors and low pH_e_, through signaling cascades that alter the ensemble of scaffolding proteins on lysosomes [5–8, 24]. In this report we demonstrated that Arl8b, through its interaction with kinesin Kif5b, is required for this response in multiple cell lines and that by depleting Arl8b, protease secretion is reduced and prostate cancer invasive growth within a 3D ECM model is greatly impaired. Impaired invasive growth in 3D culture upon Arl8b knockdown was specifically associated with decreased detection of proteolytically cleaved extracellular collagen supporting decreased release of proteases. Due to the known roles of integrins and Rho family GTPases in cancer cell invasion and the possible connection with lysosome mobility, we assayed for changes in expression and/or activity of these proteins in response to Arl8b KD. Under our experimental conditions, we did not detect differences in the levels of integrin surface expression (data not shown), nor did we detect a statistically significant change in the activation of RhoA or Rac1 in response to invasion-promoting stimuli following depletion of Arl8b. Our results from multiple assays demonstrate that Arl8b is necessary for anterograde lysosome trafficking, protease secretion, ECM degradation, and invasive motility.

Lysosomes move dynamically along microtubules and Arl8b is known to stimulate peripheral lysosome positioning through its interaction with sifA and kinesin interacting protein (SKIP) and kinesin 1 [18]. Conversely, Rab7 and its effector RILP promote retrograde traffic toward the microtubule-organizing center via recruitment of dynein motors and we recently identified Rab7 as a potential tumor suppressor through its control of lysosome positioning [8, 14]. Both Rab7 and Arl8b can simultaneously associate with the lysosome membrane and the activation of either protein can control the dynamic position of lysosomes within the cell. In some instances, both Arl8b and Rab7-dependent motor activity are simultaneously activated, resulting in lysosomal tubulation due to the opposing motor forces [22]. A recent report suggests that the lysosome population of the cell is heterogeneous and that more peripherally-localized alkaline lysosomes contain more Arl8b and less Rab7 [46]. While the mechanism regulating Arl8b and Rab7 recruitment to lysosomes remains to be defined, the activity of these two GTPases may influence lysosome fate. This intriguing notion suggests that lysosomes enriched with Arl8b may have a more secretory-like phenotype than the perinuclear Rab7-enriched lysosomes. According, we found that cells depleted of Arl8b had reduced extracellular protease activity as a result of impaired lysosome secretion.

**Figure 7:**
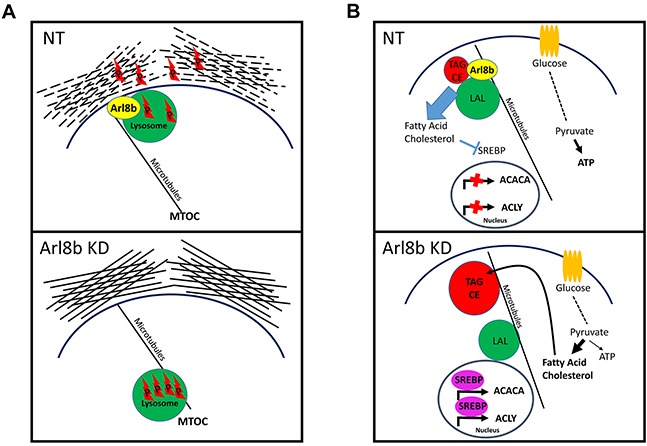
Proposed model for Arl8b regulation of protease-dependent tumor invasion and activation of the lipogenic phenotype **A.** Arl8b regulates lysosome trafficking to the periphery, which facilitates release of lysosomal proteases and ECM remodeling. P: protease. **B.** Arl8b regulates hydrolysis of internalized and/or stored lipids preventing unnecessary, metabolically inefficient *de novo* lipogenesis. This mechanism would maintain adequate ATP production supporting a proliferative capacity in a low nutrient environment. LP: lysosomal proteases, TAG: triaclyglycerides, CE: cholesterol esters, LAL: lysosomal acid lipase.

The general role of lysosomes in metabolism, in particular lipophagy of stored neutral lipids and endo-lysosomal fusion for hydrolysis of internalized neutral lipids, is rapidly evolving. Free fatty acids and cholesterol are liberated from endosomes only after hydrolysis by LAL upon interaction with lysosomes and transport through Niemann Pick proteins [3]. Liberation of these lipids suppresses SREBP transcriptional activity to prevent unnecessary *de novo* lipogenesis [47]. We have made several observations indicating a metabolic consequence of Arl8b depletion on prostate cancer growth. First, the proliferation rate of Arl8b KD cells was more greatly suppressed when serum was removed as compared to the parental line. Second, we detected a striking difference in the abundance of intracellular neutral lipid inclusion bodies. Third, our data demonstrated that Arl8b knockdown resulted in a heightened lipogenic phenotype. Together, these data strongly mirror Wolman's syndrome and Niemann Pick types C and D; conditions in which exogenous lipids are not liberated from endocytic vesicles [44]. As we observed with Arl8b KD, this results in accumulation of neutral lipids within inclusion bodies, as well as an unnecessarily heightened lipogenic phenotype. Whereas these health conditions are caused by loss of LAL or fatty acid transporters, Arl8b KD phenotypes are likely the result of impaired lysosome mobility and/or vesicle fusion [48]. Specifically, our data do not indicate a problem with glucose uptake or glycolysis necessarily. Rather, we propose that metabolism downstream of glycolysis is diverted to anabolic lipogenesis when Arl8b is knocked down (Figure 7), trumping an energy yielding catabolic path, limiting the propensity for cell division [49]. These findings highlight the essential nature of Arl8b-directed lysosome function in controlling the metabolic phenotype.

These data have demonstrated that Arl8b-directed lysosome function plays two critical roles in prostate cancer progression. We have found that: 1) during periods of growth and proliferation, Arl8b is necessary for regulating lysosome mediated metabolic processes. This function would be particularly essential in low nutrient environments, such as during early, poorly vascularized, cancer development and during late, poorly perfused dense tumor growth. In addition to this, we have determined that: 2) Arl8b is required for anterograde lysosome trafficking which occurs in response to invasion-promoting stimuli. In addition to promoting a motile phenotype, these stimuli elicit anterograde lysosome trafficking in order to degrade and reorganize ECM to allow invasive growth and ultimately vascular intravasation and metastasis. While other non-lysosomal proteases are also known to play a role in ECM degradation, it is clear from our work and others that lysosomal protease release is equally important. These results suggest Arl8b plays a critical role during periods of invasive cancer growth through control of lysosomal protease release (Figure 7).

Most strikingly, we found that prostate cancer cells depleted of Arl8b did not form subcutaneous xenograft tumors. No evidence of xenograft growth was detectable at any point during the experiments, and identically processed cells seeded in parallel in culture dishes grew normally. We find it likely that our conclusions with respect to nutrient deprived growth account for the inability to grow subcutaneously. We did not find evidence that Arl8b KD causes a reduction in the stem cell population, nor was deficiency in nutrient sensing observed by changes to mTOR signaling. However, it is possible that the proteolytic role of Arl8b is required for the initial establishment of a tumor bed in which to grow through ECM remodeling [50]. Regardless, we have provided evidence of two mechanisms for Arl8b involvement in cancer progression. Future work will further clarify the role of Arl8b-dependent lysosome function *in vivo* at different stages of cancer progression.

In conclusion, we provide data supporting a role for Arl8b as an important regulator of metabolic phenotype, protease secretion, invasion, and proliferation in multiple cancer cell lines. Most notably, we found Arl8b is essential for *in vivo* xenograft tumor growth of prostate cancer cells. Taken together, these intriguing findings support the therapeutic potential of future Arl8b-targeting and lysosome-modulating agents.

## MATERIALS AND METHODS

### Cell culture

DU145 prostate cancer (PCa) cells were purchased from ATCC (Manassas, VA). PPC1 PCa cells were originally obtained from Dr. Arthur Brothman [51]. Cells were maintained in 10% fetal bovine serum (FBS) (Gemini, West Sacramento, CA) RPMI (Cellgro, Manassas, VA). MDA MB 231 breast cancer cells were obtained from ATCC and cultured in DMEM (Cellgro) supplemented with 10% FBS. All cells were maintained in a 37°C incubator with 5% CO_2_. Cells were subcultured upon attaining 75% confluence.

### Antibodies

Immunofluorescence:Lysosome Associated Membrane Protein-1 (LAMP-1, H4A3-s) Iowa State University Developmental Hybridoma Bank 1:200; Dylight 594 Donkey anti-mouse (Jackson IR, West Grove, PA) 1:200; Arl8a/Arl8b a generous gift from Dr. Michael Brenner (Harvard) 1:500 [20]. Immunoblot: Cell Signaling Technologies (Beverly, MA) 1:1000: pMet Y1234/1235, pAKT S473, pEGFR Y845, pERK T202/Y204, p70S6K T389, CD44; Kif5b (1:1000) EMD Millipore (Darmstadt, Germany); Actin (A2066) (Sigma-Aldrich, St. Louis, MO) 1:5000; Tubulin (NeoMarkers, Fremont, CA) 1:20000; EGFR (Santa Cruz Technologies, Dallas, TX), c-Met and Rac1 (Life Technologies, Carlsbad, CA) 1:1000; RhoA (Cytoskeleton, Denver, CO) 1:500.

### Immunoblot

Whole cell lysates were taken in boiling Laemmli buffer (0.125 M Tris-HCL, pH 6.8, 4% SDS, 0.13 mM bromophenol blue, 1M sucrose, and 2% 2-mercaptoethanol) and boiled 5 minutes. Lysates were run on polyacrylamide gels and transferred onto PVDF. Membrane was blocked in 5% milk TBST (20 mM Tris, 137 mM NaCl, 0.1% Tween 20, pH 7.5) and probed with the indicated primary antibodies overnight at 4°C followed by HRP-conjugated secondary antibodies (GE Healthcare, Pittsburgh, PA) for 1 hour. Pierce ECL 2 (Life Technologies) was used for chemiluminescent detection on x-ray film.

### Proliferation assay

Cells were seeded to approximately 15% confluency prior to proliferation analysis in complete growth media (with serum), or serum-free media at 100%, 30%, 10%, or 3% of normal glucose concentration. Images were taken every 4 hours with the IncuCyte ZOOM imaging system (Essen Bioscience, Ann Arbor, MI). Relative change in confluency over 4 days was determined using the IncuCyte ZOOM analysis software. Conditions were tested in quadruplicate for three independent experiments.

### Lysosome analysis

As previously described [6]. Briefly, the LysoTracker software (a generous gift from Meiyappan Solaiyappan at Johns Hopkins University) was used to analyze the distance of fluorescently-labeled lysosomes from the nucleus border. A total of 25 cells from each experimental condition were analyzed. Significance was determined using Two-Tailed Mann-Whitney T test in GraphPad 3.0 software.

### Lentivirus delivery of shRNA

Scrambled non-target (NT) shRNA (SHC202V) or Arl8b shRNA was delivered to DU145 and MDA MB 231 (i:TRCN0000072857; ii: TRCN0000072854) or PPC1 (i:TRCN0000072854; ii: TRCN0000072853) cells using Mission Lentivirus Transduction particles (Sigma). Cells were plated at 50% confluence in 6 well plates. Cells were treated with 6 μg/mL polybrene (Millipore) and lentivirus for 48 hours then selected with 1.8 μg/mL puromycin (Calbiochem, Billerica Massachusetts). NT and Arl8b KD cells were maintained under puromycin selection for the duration of the experiments.

### Transwell motility assays

2×10^4^ cells were plated on top of transwell inserts with an 8 μm pore size in serum-free RPMI. Complete media or complete media plus 2×10^4^ NT cells was plated on the bottom of the insert. Cells migrated toward the underside of the insert for 48 hours. Inserts were then fixed with 4% paraformaldehyde (PFA) for 20 minutes at room temperature and then stained with 0.1% crystal violet for 20 minutes. Cells remaining on the top side of the insert were removed using a cotton swab. Cells that migrated to the underside of the insert were imaged at 10X using an Olympus CKX41 microscope and DPManager software. Five fields from three independent experiments were collected and representative images are shown. The number of motile cells was counted.

### Microscopy

Cells were seeded on glass coverslips for all experiments. Cells were fixed in ice-cold 4% PFA (Sigma) for 20 minutes followed by one wash with 1X PBS. Cells were then incubated at room temperature for one hour with LAMP-1 diluted 1:200 in BSP (0.25% BSA and 0.1% Saponin in PBS), washed twice with PBS and then incubated with 1:100 Dylight 594 anti-mouse in BSP for one hour at room temperature. Phalloidin 488 (Molecular Probes) was applied at 1:200 in BSP for 20 minutes at room temperature. Slides were washed three times in PBS and mounted with DAPI containing SlowFade gold reagent (Invitrogen S36938). Images were taken on an Olympus BX-50 microscope using MetaView software. To assess cell scattering, cells were stained with 1:100 Phalloidin:BSP for 20 minutes and 10X images were acquired using a Nikon Eclipse TE300 inverted epifluorescence microscope. Neutral lipids were stained with 100 nM Nile Red (Acros Organics) for 5 minutes at room temperature after PFA fixation. 3D cultures and neutral lipid inclusion bodies were imaged at 63X using a Leica TCS SP5 confocal microscope. Quantitation of neutral lipid staining was performed using ImageJ software on five imaging fields from three independent experiments and representative images are presented.

### Quantitative reverse transcriptase PCR

Cells were seeded to 70% confluency in 10 cm dishes in complete growth media. Cells were collected in Trizol (Invitrogen) and RNA was isolated according to manufacturer's instructions. The SuperScript First-Strand kit (Invitrogen) was used for cDNA synthesis. RT^2^ SYBR Green Flour FAST Mastermix (Qiagen; Valencia, CA) was used in qPCR reactions. Cycling conditions were 95°C 10 minutes, followed by 40 cycles of 95°C 10 seconds and 55°C 30 seconds. Melt curves were assessed for each run and reverse transcriptase negative and template negative controls were used. Reactions were carried out using a Bio-Rad CFX96 Real-Time PCR detection System with Bio-Rad CFX Manager 3.0 software. Primers were designed using Integrated DNA Technologies (Coralville, IA) PrimerQuest software and sequences used include (5′ to 3′): ACACA forward: CTTGTCACCTGCTTCTGT, ACACA reverse: AGACATGCTGGACCTTATG; ACLY forward: TTCGGCAGAGACAGGTA, ACLY reverse: GAGGTGGTACAGATGAACTT; GAPDH forward: GTCGGAGTCAACGGATTT, GAPDH reverse: AGTTGAGGTCAATGAAGGG.

### Surface biotinylation

Cells were grown to 70% confluency in 10 cm dishes. Cells were washed once with 4°C PBS to halt membrane internalization. Surface-exposed proteins were biotinylated with 0.1 mg/mL EZ-Link biotin (Invitrogen) in PBS at 4°C for 15 minutes. Excess biotin was quenched and removed with three 5-minute washes of 100 mM glycine in PBS at 4°C. Cell lysates were taken in NP-40 lysis buffer (25 mM Tris-HCl, pH 7.4, 150 mM NaCl, 1 mM EDTA, 1% NP-40 and 5% glycerol) containing protease inhibitor cocktail (Roche) at 4°C. Lysates were incubated overnight at 4°C with end-over-end rotation followed by removal of debris by centrifugation at 13,000xg for 10 minutes. Protein concentrations were determined and biotin pulldown was performed on 50 μg of protein in equal final volume of NP-40 lysis buffer using 50 μl of washed streptavidin-conjugated sepharose beads (GE Healthcare). Samples were rotated end-over-end overnight at 4°C. Biotinylated protein was isolated and washed (70 volumes) by repeated centrifugation at 5,000xg for 30 seconds. Protein was eluted in 30 μl of Laemmli buffer. Samples from two independent experiments for each cell line were analyzed by immunoblot and representative results are shown.

### 3D culturing

3D culturing was performed as previously described with slight modifications [27, 52]. Matrigel (BD Bioscience) supplemented with 25 μg/mL DQ-Collagen IV (Molecular Probes) was applied to glass coverslips and allowed to solidify at 37°C for 30 minutes. 1×10^5^ cells were seeded on top of the 3D matrix in 2 mL complete media. Cells were allowed to settle for 2 days prior to treatment with either 100 ng/mL EGF or 33 ng/mL HGF for 2 additional days. To terminate the experiment, the culture was fixed for 30 minutes in warm 4% PFA at 37°C. Actin was then stained using 1:200 Phalloidin 633 (Molecular Probes) for 30 minutes in warm BSP at 37°C. Three fields from three independent experiments were imaged and representative images are shown. To quantify cleaved DQ-collagen IV signal, the area of the actin cytoskeleton was subtracted from the DQ-collagen IV fluorescent signal using Image Calculator (ImageJ). Remaining extracellular DQ-collagen IV fluorescence was assessed by integrated density (ImageJ) and displayed as arbitrary units.

### RhoA and Rac1 activation assays

DU145 NT or Arl8b KD cells were plated in a 10 cm dish and allowed to grow to 70% confluence. Cells were serum starved for 18 hours and then treated with 100 ng/mL EGF for 5 minutes. Active Rac1 was assessed using the Rac1-GTPase pull-down assay kit (Thermo Scientific) and active RhoA was assessed using the RhoA pull-down activation assay biochem kit (Cytoskeleton). Pull-down assays were performed according to the manufacturer protocol. Densitometry of at least 3 representative immunoblots was performed to assess significance.

### Xenograft assay

PPC1 PCa cells were transduced with NT or Arl8b shRNA. Stable knockdown of Arl8b was confirmed by immunoblot prior to the start of the *in vivo* experiment. Cells were collected and re-suspended to 1×10^6^ cells/100 μl in HBSS and were injected subcutaneously into the right hind flank of male SCID/bg mice aged 6-10 weeks. Seventeen mice per experimental condition were used for a total of 34 mice per experiment. Tumor volume (mm^3^) was measured twice-weekly using a caliper and was calculated as L (largest dimension) × W^2^. At the conclusion of the experiment, tumors were harvested and weighed. Tumor portions from each mouse were formalin fixed or frozen for further analysis. Two independent xenograft experiments were performed. All experiments were approved by the LSUHSC-S Institutional Animal Care and Use Committee.

### Statistical analysis

Data are expressed as means ± SEM. Statistical analysis was performed using Mann-Whitney T Test (two-tailed) with a minimum *P*-value <0.05 as significant, unless otherwise stated.

## SUPPLEMENTARY FIGURES AND MOVIE





## Abbreviations

pH_e_: extracellular pH
ECM: extracellular matrix
MMP: matrix metalloproteinase
MTOC: microtubule organizing center
RILP: Rab-interacting lysosomal protein
Arl8b: ADP-Ribosylation Factor like Protein 8b
PCa: prostate cancer
NT: non-target
KD: knockdown
EGF: epidermal growth factor
HGF: hepatocyte growth factor
JLA: juxtanuclear lysosome aggregation
LAMP-1: Lysosome Associated Membrane Protein-1
LAL: Lysosomal Acid Lipase
mTOR: Mammalian Target of Rapamycin
mTORC1: mTOR Complex 1
TME: Tumor Microenvironment
SREBP: Sterol Regulatory Element-Binding Protein
