# Supplementary material for: The Arf-like GTPase Arl8b is essential for three-dimensional invasive growth of prostate cancer in vitro and xenograft formation and growth in vivo
Source: Oncotarget. 2016 Apr 18;7(21):31037–52. doi: 10.18632/oncotarget.8832 (PMC5058737; doi:10.18632/oncotarget.8832)
Supplement: Supplementary file 1 [file oncotarget-07-31037-s001.pdf]

SUPPLEMENTARY FIGURES

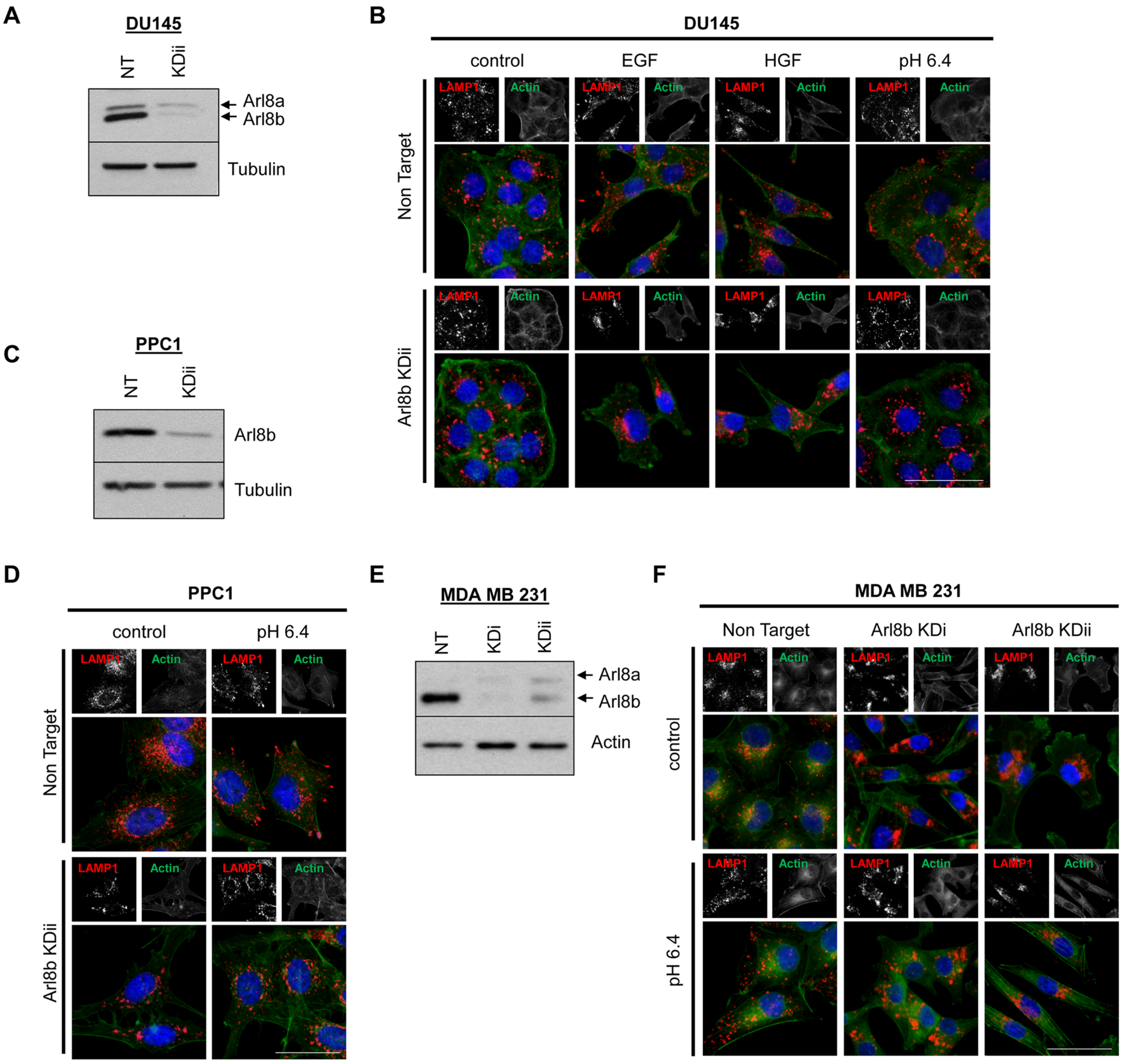

Supplementary Figure S1: Arl8b knockdown prevents anterograde lysosome trafficking in multiple cancer cell lines. **A.** DU145 cells were transduced with lentiviral-delivered shRNA sequences targeted to Arl8b (KDii) or non-targeted (NT) shRNA. Immunoblot confirms knockdown. **B.** DU145 cells were treated with low pH for 2 hours or 33 ng/mL HGF or 100 ng/mL EGF for 18 hours, then fixed, stained for LAMP-1 (Red), phalloidin (Green), and DAPI (Blue), N=3. **C.** PPC1 cells were transduced with lentiviral-delivered shRNA sequences target to Arl8b (ii) or non-targeted shRNA. Immunoblot confirms knockdown. **D.** PPC1 cells were treated with low pH for 2 hours, then fixed, stained for LAMP-1 (Red), phalloidin (Green), and DAPI (Blue), N=3. **E.** MDA MB 231 cells were transduced with lentiviral-delivered shRNA sequences targeted to Arl8b (KDi or KDii) or non-targeted (NT) shRNA. Immunoblot confirms knockdown. **F.** MDA MB 231 cells were treated with low pH for 2 hours, then fixed, stained for LAMP-1 (Red), phalloidin (Green), and DAPI (Blue), N=3.

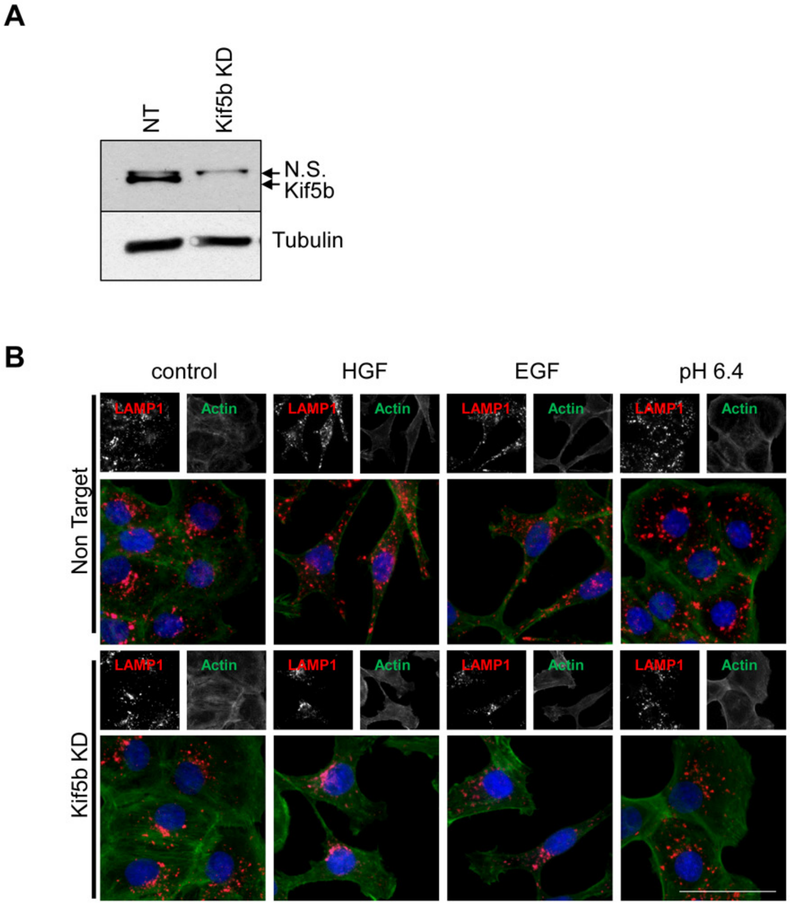

Supplementary Figure S2: Kif5b is necessary for anterograde lysosome trafficking. A. DU145 cells were transduced with lentiviral-delivered shRNA targeting kif5b (KD). Immunoblot confirms knockdown. **B.** DU145 Non Target or Kif5b KD cells were treated with low pH for 2 hours or 33 ng/mL HGF or 100 ng/mL EGF for 18 hours, then fixed, stained for LAMP-1 (Red), phalloidin (Green), and DAPI (Blue), N=3.

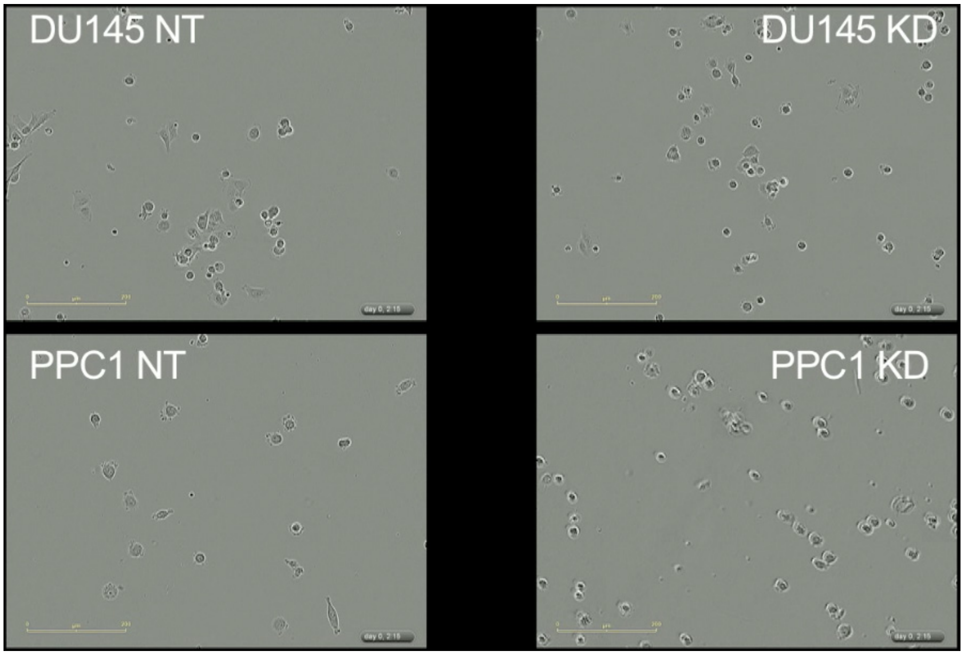

Supplementary Figure S3: Arl8b KD cells display slower cell spreading compared to NT cells. DU145 and PPC1 NT and Arl8b KD cells were seeded on tissue culture treated plastic in complete media. Cells were imaged every 15 minutes using the IncuCyte ZOOM imaging system.

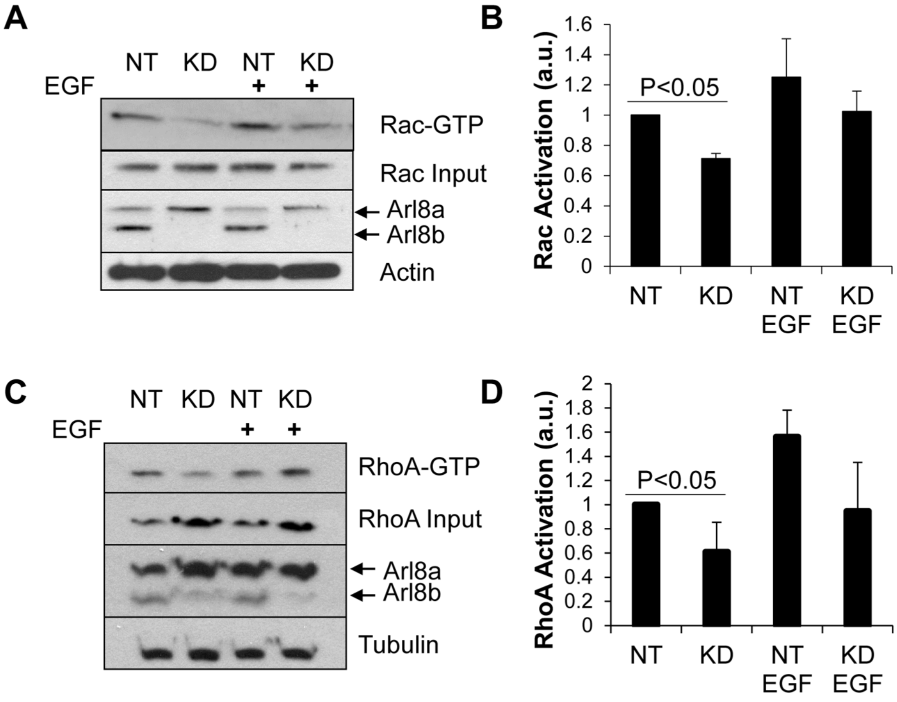

Supplementary Figure S4: Arl8b knockdown results in reduced Rac1 and RhoA activation. A. DU145 Non Target or Arl8b KD cells were treated with 100 ng/mL EGF for 5 minutes. Active Rac1 pull down assay was performed with fresh whole cell lysates and samples were immunoblotted for the indicated proteins. **B.** Represents densitometry from 4 individual active Rac1 pull down assays. Data are shown as mean ± SEM. **C.** DU145 Non Target or Arl8b KD cells were treated with 100 ng/mL EGF for 5 minutes. Active RhoA pull down assay was performed with fresh whole cell lysates and samples were immunoblotted for the indicated proteins. **D.** Represents densitometry from 7 individual active RhoA pull down assays. Data are shown as mean ± SEM.

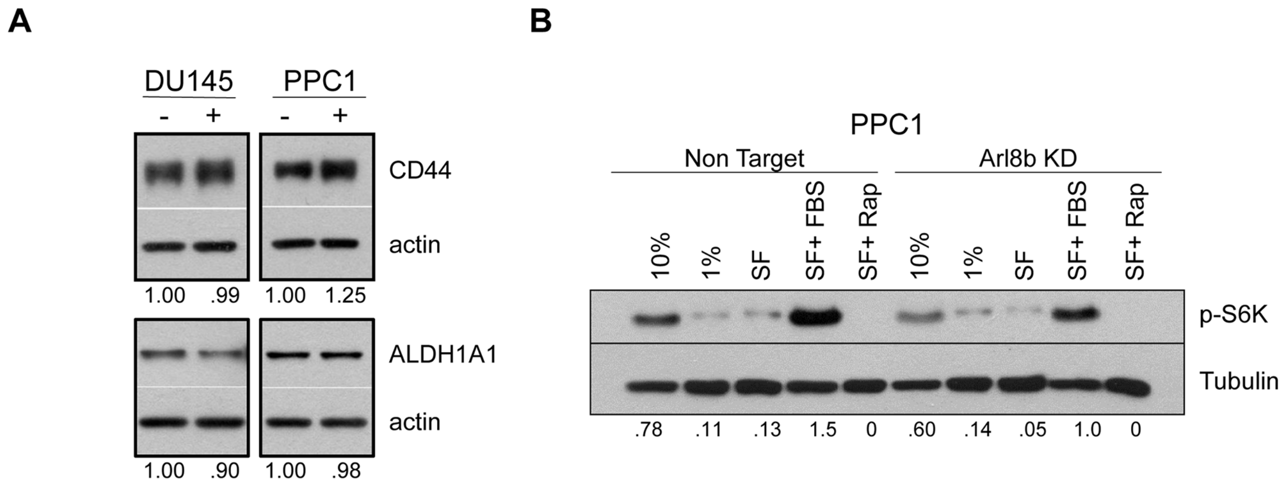

Supplementary Figure S5. Arl8b KD cells do not have reduced stemness or altered mTOR-mediated nutrient sensing. **A.** Whole cell lysates from NT or Arl8b KD cells in the indicated parental cell lines were collected and probed for the indicated proteins by immunoblot. Densitometric analysis of CD44 or ALDH1A1 relative to Tubulin is shown. **B.** PPC1 NT or Arl8b KD cells were treated with a range of serum concentrations or 1 μM Rapamycin for 8 hours. Whole cell lysates were collected and analyzed by immunoblot. Densitometric analysis of p-S6K relative to Tubulin is shown.
